# Prevalence of Novel Myositis Autoantibodies in a Large Cohort of Patients with Interstitial Lung Disease

**DOI:** 10.3390/jcm9092944

**Published:** 2020-09-11

**Authors:** Sofia A. Moll, Mark G. J. P. Platenburg, Anouk C. M. Platteel, Adriane D. M. Vorselaars, Montse Janssen Bonàs, Claudia Roodenburg-Benschop, Bob Meek, Coline H. M. van Moorsel, Jan C. Grutters

**Affiliations:** 1 ILD Center of Excellence, Department of Pulmonology, St. Antonius Hospital, Post box 2500, 3435 CM Nieuwegein, The Netherlands; m.platenburg@antoniusziekenhuis.nl (M.G.J.P.P.); a.vorselaars@antoniusziekenhuis.nl (A.D.M.V.); m.janssenbonas@antoniusziekenhuis.nl (M.J.B.); c.benschop@antoniusziekenhuis.nl (C.R.-B.); c.van.moorsel@antoniusziekenhuis.nl (C.H.M.v.M.); j.grutters@antoniusziekenhuis.nl (J.C.G.); 2 Department of Medical Microbiology and Immunology, St. Antonius Hospital, 3435 CM Nieuwegein, The Netherlands; a.platteel@antoniusziekenhuis.nl (A.C.M.P.); b.meek@antoniusziekenhuis.nl (B.M.); 3 Division Heart & Lungs, University Medical Centre Utrecht, 3435 CM Utrecht, The Netherlands

**Keywords:** interstitial lung disease, connective tissue disease, idiopathic interstitial pneumonia, myositis antibody, anti-Ks, anti-Ha, anti-Zoα, anti-cN1A

## Abstract

Connective tissue diseases (CTDs) are an important secondary cause of interstitial lung disease (ILD). If a CTD is suspected, clinicians are recommended to perform autoantibody testing, including for myositis autoantibodies. In this study, the prevalence and clinical associations of novel myositis autoantibodies in ILD are presented. A total of 1194 patients with ILD and 116 healthy subjects were tested for antibodies specific for Ks, Ha, Zoα, and cN1A with a line-blot assay on serum available at the time of diagnosis. Autoantibodies were demonstrated in 63 (5.3%) patients and one (0.9%) healthy control (*p* = 0.035). Autoantibodies were found more frequently in females (*p* = 0.042) and patients without a histological and/or radiological usual interstitial pneumonia (UIP; *p =* 0.010) and a trend towards CTD-ILDs (8.4%) was seen compared with other ILDs (4.9%; *p* = 0.090). The prevalence of antibodies specific for Ks, Ha, Zoα, and cN1A was, respectively, 1.3%, 2.0%, 1.4%, and 0.9% in ILD. Anti-Ha and Anti-Ks were observed in males with unclassifiable idiopathic interstitial pneumonia (unclassifiable IIP), hypersensitivity pneumonitis (HP), and various CTD-ILDs, whereas anti-cN1A was seen in females with antisynthetase syndrome (ASS), HP, and idiopathic pulmonary fibrosis (IPF). Anti-Zoα was associated with CTD-ILD (OR 2.5; 95%CI 1.11–5.61; *p* = 0.027). In conclusion, a relatively high prevalence of previously unknown myositis autoantibodies was found in a large cohort of various ILDs. Our results contribute to the awareness that circulating autoantibodies can be found in ILDs with or without established CTD. Whether these antibodies have to be added to the standard set of autoantibodies analysed in conventional myositis blot assays for diagnostic purposes in clinical ILD care requires further study.

## 1. Introduction

Interstitial lung diseases (ILDs) are a heterogeneous group of diffuse parenchymal lung disorders, characterized by inflammation or fibrosis of the pulmonary interstitium. ILDs can be idiopathic or secondary to known causes including connective tissue diseases (CTDs) [1,2,3,4,5]. It is challenging to distinguish CTD-ILD from other ILDs as clinical, functional, radiological, and pathological characteristics can be similar [6]. Moreover, an interstitial pneumonia (IP) may be the first or single clinical manifestation of an underlying CTD [4,6]. In general, outcomes on treatment response to immunosuppressive therapy and survival are better in CTD-ILD compared to the majority of ILDs without established CTD [5,6,7,8,9,10]. Thus, discriminating these conditions in the diagnostic work-up is essential.

Serologic testing for autoantibodies by a myositis blot is recommended in pulmonary fibrosis suspected for an underlying CTD, which includes myositis specific antibodies (MSA) and myositis associated antibodies (MAA) [1,3,4,6,11,12,13,14,15,16]. MSA and MAA are found in patients with idiopathic interstitial myopathies but also occur in patients with rheumatic diseases including CTD-ILDs [3,4,5,6,11,12,13,14,15,16,17,18,19,20,21,22,23]. Moreover, myositis antibody positivity has been described in other ILDs, including hypersensitivity pneumonitis (HP) and idiopathic IPs [11,16,23,24,25]. However, many suspected patients are negative for the standard set of autoantibodies. Here, we focus on relatively unknown antibodies, including antibodies specific for asparaginyl-t-RNA synthetase (anti-Ks), tyrosyl-t-RNA synthetase (anti-Ha), phenylanyl-t-RNA synthetase alpha (anti-Zoα), and cytosolic-5-nucleotidase-1A (anti-cN1A). Circulating antibodies specific for Ks, Ha, and cN1A have been described in inclusion body myositis (IBM), systemic sclerosis (Ssc), and Sjögren’s syndrome, whereas anti-Zo has been identified in anti-synthetase syndrome (ASS) with ILD [26,27,28,29,30,31,32,33,34,35,36]. However, data are scarce on the prevalence and associations of these autoantibodies in CTD-ILD and other ILD.

The aim of this study was to evaluate the prevalence of antibodies to Ks, Ha, Zoα, and cN1A in patients with CTD-ILD compared to various other ILDs and healthy controls, measured by a line blot assay. Clinical characteristics of ILD patients with autoantibody positivity are described.

## 2. Experimental Section

### 2.1. Patient Selection

A retrospective cohort study was conducted at the St Antonius ILD Center of Excellence Nieuwegein, a tertiary ILD referral center in the Netherlands. The majority of patients were diagnosed between 2000 and 2019 with an ILD with and without established CTD. Serum collected at the date of diagnosis was evaluated for the presence of autoantibodies by a research myositis line-blot assay. Furthermore, sera of healthy, non-ILD blood donors were screened for autoantibodies and compared with ILD patients.

Diagnosis of ILD was assessed according to official recommendations of the American Thoracic Society/European Respiratory Society in a multidisciplinary discussion with an ILD pulmonologist, experienced thoracic radiologist, and a pathologist, when required [37]. All patients with pulmonary fibrosis were screened for an underlying CTD by the chest physician and referred to the rheumatologist for further diagnostic work-up if a CTD was suspected.

Patients were classified as having a diagnosis of CTD-ILD or ILD without established CTD (non-CTD-ILD). Patients were checked for any revisions of the ILD diagnosis during two years of follow-up, as an IP can occur two years before an associated CTD [3,6]. CTD-ILDs included antisynthetase syndrome (ASS), Sjögren’s syndrome, rheumatoid arthritis associated ILD (RA-ILD), systemic sclerosis (Ssc), dermatomyositis (DM), polymyositis (PM), immune mediated necrotizing myopathy (IMNM), inclusion body myositis (IBM), overlap myositis, systemic lupus erythematosus (SLE), mixed CTD, and other CTD-ILD. Non-CTD-ILDs included idiopathic pulmonary fibrosis (IPF), hypersensitivity pneumonitis (HP), unclassifiable idiopathic interstitial pneumonia (Unclassifiable IIP), non-specific interstitial pneumonia (NSIP), cryptogenic organizing pneumonia (COP), pneumoconiosis, drug-induced IP, and other ILD.

The baseline characteristics of patients with ILD tested for novel autoantibodies were described. This also included pulmonary function tests (PFTs), which were performed according to ERS recommendations. Furthermore, baseline characteristics on high-resolution computed tomography (HRCT) and in lung biopsies (when available) were described and classified according to the most recent American Thoracic Society/European Respiratory Society recommendations as a pattern of usual interstitial pneumonia (UIP), probable UIP, indeterminate UIP, or alternative diagnosis [38]. Moreover, the prevalence of patients meeting the non-serological criteria for interstitial pneumonia with autoimmune features (IPAF) was evaluated [39]. In addition, the presence of antinuclear antibodies (ANA) at baseline, PFT change after one year (expressed as an absolute delta positive or negative change), and survival outcomes were described.

The study was approved by the St Antonius institutional review board under protocol number 842002003 and patients provided written informed consent for research purposes.

### 2.2. Determination of Antibodies

In the current study, we evaluated the prevalence of novel myositis antibodies as measured by a blot assay. Detection of antibodies by a line blot assay is concordant with the analysis by the gold standard, immunoprecipitation, and specific ELISAs [40]. Therefore, we did not perform a method comparison between this line blot assay and other tests. Antibodies were detected in serum using a line blot assay (EUROLINE Myositis Research Profile, EUROIMMUN, Lübeck, Germany) in collaboration with Biognost, Kortrijk, Belgium. To date, this blot has been used for research purposes. Stability (stress test and real-time test including transport stability), reproducibility, interferences, serum/plasma comparison, and cross reactivity comply with CE standard for CE-certification of antibody testing against the concerned antibodies. This blot was run between 05-2019 and 07-2019 and identifies antibodies specific for asparaginyl-transfer-RNA synthethase (anti-Ks), tyrosyl transfer-RNA synthethase (anti-Ha), phenylanyl-transfer-RNA synthethase alpha (anti-Zoα) and specific for cytosolic-5-nucleotidase-1A (anti-cN1A). Analysis of the immunoblot strips was performed with the EUROLINEScan software (EUROIMMUN, Lübeck, Germany) according to manufacturer’s recommendations as described for the EUROLINE Autoimmune Inflammatory Myopathies line blot assay. Staining strips were qualified as either negative (-), weakly positive (+) and positive (++), which corresponds with intensity levels of 0–10, 11–25, and >25 respectively. Antibody reactivity on a combined weak positive with positive intensity level and on a positive intensity level only was separately evaluated. For further details, we refer to the methods and materials section of the study of Platteel et al. [21].

### 2.3. Statistical Analysis

Baseline characteristics were expressed as numbers and percentages or mean and standard deviation, depending on the type of data. Continuous and categorical variables were tested with a student’s T-test/one-way ANOVA and Chi-square test/Fisher’s exact test respectively. The statistical analysis was performed by software IBM SPSS Statistics for Windows version 24.0, IBM Corp, Armonk, NY, USA. A *p*-value less than 0.05 was considered as statistically significant. Graphs were drafted in GraphPad Prism version 8.3 for Windows, GraphPad Software, San Diego, CA, USA. 

## 3. Results

### 3.1. Baseline Characteristics

A total of 131 patients with CTD-ILD and 1063 patients with non-CTD-ILD were included in this study (Table 1). Age and ANA positivity were statistically different between CTD-ILD and non-CTD-ILD patients. Further classification of baseline characteristics per ILD diagnosis can be found in the supplementary data (Supplementary Tables S1 and S2).

### 3.2. Prevalence of Antibodies in ILD and Healthy Controls

Antibody prevalence of novel myositis autoantibodies was evaluated for all ILD, 116 healthy controls (Table 2) and per ILD diagnosis (Supplementary Tables S3 and S4). Regarding the antigens that stained ‘positive’, a total of 63 ILD patients (5.3%) demonstrated antibody reactivity, which was significantly higher compared to healthy controls (0.9%; *p* = 0.035; Table 2). The prevalence of antibody reactivity against myositis antibodies altogether on combined positive and weakly positive levels was also higher in ILD patients (10.0%) compared to healthy controls (2.6%; *p* = 0.009; Table 2). Anti-Ha was the most prevalent antibody found in ILD (2.0%), followed by anti-Zoα (1.4%) and anti-Ks (1.3%). In healthy controls, antibody reactivity at a positive level was observed in only one subject (0.9%; anti-cN1A). Prevalence of anti-Zoα reactivity on combined positive and weakly positive levels was significant higher in CTD-ILD compared to non-CTD-ILD (*p* = 0.047). Prevalence per antibody was not significantly different between all ILD patients and healthy subjects (Table 2), nor between the ILD subgroups.

### 3.3. Antibody Positive ILD Versus Antibody Negative ILD

Patients with antibody reactivity at intensity level ‘positive’ only were compared to patients without any antibody reactivity (Table 3). Patients with antibody reactivity on a ‘weak positive’ intensity level only were first excluded from this analysis (n = 56). Antibody positive subjects were more often females (47.6%) compared to antibody negative subjects (34.9%; *p* = 0.042). Furthermore, antibody positive ILD was less frequently characterized by a pattern of UIP in the biopsy (11.1%) compared to antibody negative ILD (35.8%; *p* = 0.032). Moreover, a trend towards absence of the UIP pattern on HRCT in antibody positive subjects was present (22.0%) compared to antibody negative subjects (30.9%; *p* = 0.158). Altogether, antibody positive subjects demonstrated less frequently a UIP pattern on either HRCT or in lung biopsies (15.9%) compared to antibody negative ILD as well (36.6%; *p* = 0.010). No differences were found for age, ANA positivity, or baseline PFT (Table 3). Additionally, a three-way analysis comparing antibody positive ILD patients with antibody weak positive ILD and antibody negative ILD was performed. Significantly more females (*p* = 0.049) and fewer patients with UIP patterns on either HRCT or in lung biopsies (*p* = 0.016) were also observed in the antibody positive ILD group compared to antibody weak positive ILD and antibody negative ILD.

Next, follow-up characteristics were evaluated for antibody positive ILD compared to antibody negative ILD. PFT change values were available in 36 antibody positive ILD and 678 antibody negative ILD. No significant differences were found in FVC change between antibody positive ILD (mean delta +2.0% pred; SD 12.3) compared to antibody negative ILD (mean delta –1.1% pred; SD 11.9; *p* = 0.145). Similarly, differences in DLCO change were not statistically significant as well between the antibody positive ILD group (mean delta –0.5% pred; SD 9.6) compared to antibody negative ILD group (mean delta –1.7% pred; SD 8.7; *p* = 0.481). Survival analysis of the groups showed no differences between antibody positive ILD (mortality rate n = 26 (41.3%); median 38.6 months: IQR 22.9–70.4) and antibody negative ILD (mortality rate n = 529 (46.8%); median 31.6 months; IQR 18.6–56.0; *p* = 0.072).

### 3.4. CTD-ILD Patients Versus non-CTD-ILD Patients

Overall, CTD-ILD patients were significantly younger compared with other ILDs (*p* < 0.001; Table 1). Furthermore, more females were observed in CTD-ILD (44.2%) compared with non-CTD-ILD (34.1%; *p* = 0.022). CTD-ILDs were frequently characterized by a radiological or histological pattern indeterminate for UIP, whereas a UIP pattern was more observed in non-CTD-ILDs, particularly in IPF (see Supplementary Tables S1 and S2). A trend towards a higher prevalence of antibody reactivity was observed in CTD-ILD (8.4%) compared with other ILD (4.9%; *p* = 0.090). In both CTD-ILD and non-CTD-ILD, anti-Ha was the most prevalent antibody found (respectively 3.1% and 1.9%), followed by anti-Zoα and anti-Ks (Table 2). Patients with an IPF, unclassifiable IIP, HP and NSIP showed cN1A antibodies (range 0.3–1.5%). On the contrary, only two CTD-ILD patients (ASS) had anti-cN1A (Supplementary Tables S3 and S4). Reactivity against multiple antigens was rare and only occurred in Sjögren’s syndrome (n = 1; anti-Ks and anti-Zoα), ASS (n = 1; anti-cN1A and anti-Zoα) and unclassifiable IIP (n = 1; anti-Ha, anti-Ks and anti-Zoα). Patients with COP revealed no reactivity against the tested antigens. ILD patients who met the non-serological IPAF criteria (n = 11, all diagnosed as unclassifiable IIP) did not show antibody reactivity against the four tested antibodies on a positive or weakly positive level. PFT change values were available in 89 CTD-ILD and 625 non-CTD-ILD patients. Non-CTD-ILD patients demonstrated significantly more FVC declines (mean delta –1.7% pred; SD 11.7) compared to CTD-ILD patients (mean delta +4.1% pred; SD 12; *p* < 0.001). Similarly, DLCO declines were more observed in non-CTD-ILD patients (mean delta –2.2% pred; SD 8.4) compared to CTD-ILD patients (mean delta +2.8% pred; SD 10.1; *p* < 0.001). Survival was significantly better in CTD-ILD patients (mortality rate n = 42 (32.1%); median 54.0 months; IQR 33.8-84.1) compared to non-CTD-ILD patients (mortality rate n = 513 (48.3%); median 30.2 months; IQR 18.1–52.4; *p* < 0.001).

### 3.5. Characteristics per Myositis Antibody

#### 3.5.1. Characteristics of anti-Ha Positive ILD

**Figure 1:**
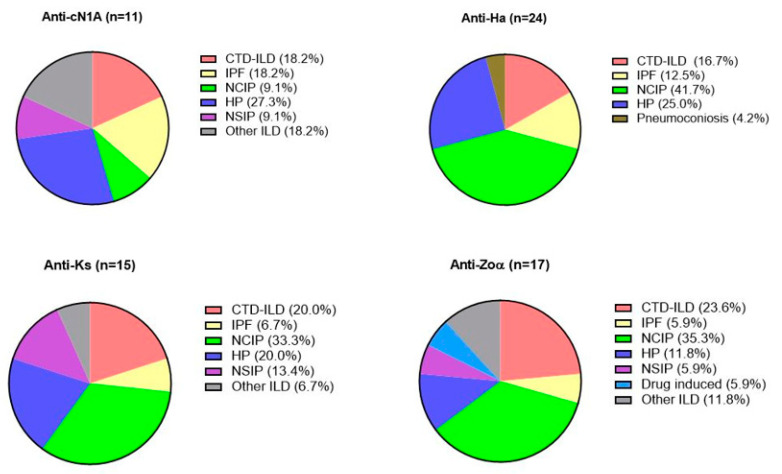
Prevalence of connective tissue disease related interstitial lung disease (CTD-ILD), idiopathic pulmonary fibrosis. (IPF), unclassifiable idiopathic interstitial pneumonia (unclassifiable IIP), hypersensitivity pneumonitis (HP), non-specific interstitial pneumonia; (NSIP), pneumoconiosis, drug induced interstitial pneumonia and other ILD, illustrated for each antibody with reactivity on a positive level.

Anti-Ha reactivity was observed in 24 patients who were classified as unclassifiable IIP (41.7%), HP (25.0%) or CTD-ILD (16.7%; Table 4, Figure 1). The CTD-ILDs consisted of ASS (n = 1), Ssc (n = 1) and Sjögren’s syndrome (n = 2, Supplementary Table S3). Alternative patterns (other than UIP subcategories) were predominately seen on HRCT (34.8%) and in histopathological lung biopsies (75%, see Table 4; per ILD diagnosis see Supplementary Tables S3 and S4).

#### 3.5.2. Characteristics of anti-Ks Positive ILD

Fifteen patients had anti-Ks antibodies, classifications including unclassifiable IIP (33.3%), HP (20.0%), and one patient with a desquamative interstitial pneumonia (DIP, 18.2%) (Figure 1). Anti-Ks positive CTD-ILDs (20.0%) consisted of patients with IBM (n = 1), RA-ILD (n = 1) and Sjögren’s syndrome (n = 1; Supplementary Table S3). A variable palette of radiological patterns was observed, whereas available lung biopsies (n = 6) showed predominately alternative patterns (66.7%, see Table 4; per ILD diagnosis see Supplementary Tables S3 and S4.

#### 3.5.3. Characteristics of anti-Zoα Positive ILD

Seventeen patients had Zoα antibodies and were predominately male (64.7%) with a smoking history (75.0%; Table 4). Antibodies were seen in unclassifiable IIP (35.3%), HP (11.8%), and CTD-ILDs (23.6%), the latter groups consisting of ASS (n = 2), DM (n = 1) and Sjögren’s syndrome (n = 1; Supplementary Table S3) patients. Zoα antibodies were found as well in DIP (n = 1), drug induced IP (flecainide-induced, n = 1) and combined pulmonary fibrosis and emphysema (CPFE, n = 1). Non-UIP radiological patterns were mostly observed. Available lung biopsies, all from non-CTD-ILDs, demonstrated a histological pattern alternative for UIP (Table 4; Supplementary Tables S3 and S4).

#### 3.5.4. Characteristics of anti-cN1A Positive ILD

Eleven subjects showed anti-cN1A reactivity and were predominately female (81.8%, Table 4). As illustrated in Figure 1, patients included HP (27.3%), IPF (18.2%), or CTD-ILD (18.2%, all with ASS). cN1A antibodies were found as well in smoking-related IP (SR-ILD) and respiratory bronchiolitis IP (RB-ILD). Almost half of anti-cN1A positive ILD subjects showed a radiological UIP pattern (40%), which was significantly higher compared to the other antibody groups (*p* = 0.048). Concerning histological patterns, a variable palette of patterns was observed in these patients (Table 4 and Supplementary Tables S3 and S4),

### 3.6. Associations Between Antibodies and ILD

A logistic regression analysis was performed to evaluate associations between staining intensity levels and ILD classification. Antibody Zoα was found to be associated with CTD-ILD compared to other ILD when the “weakly positive” and “positive” groups were combined (OR 2.5; 95% CI 1.11–5.61; *p* = 0.027, Table 5). Further analysis demonstrated that the association was strongest for CTD-ILD compared to IPF within the group qualified as ‘positive’ (OR 9.6: *p* = 0.044).

## 4. Discussion

In this explorative study, we described the prevalence and clinical characteristics of a novel set of myositis related autoantibodies in a large cohort of patients with ILD. The pooled analysis showed that the prevalence of antibodies specific for Ha, Ks, Zoα, and cN1A was significantly higher in ILD compared to healthy controls. Antibodies specific for Ha, Ks, and Zoα were observed in unclassifiable IIP, HP, and various CTD-ILDs, whereas cN1A antibodies were seen predominately in female subjects with ASS, HP, and IPF. Furthermore, anti-Zoα was associated at a weakly positive and positive level with CTD-ILD compared to other ILD. In patients with circulating autoantibodies, radiological and/or histological non-UIP patterns on HRCT and/or in histological lung biopsies were predominately seen.

To date, little is known about the presence of these myositis antibodies in CTD-ILD and other ILD. Our study provides novel data on prevalence and clinical features of relatively unknown myositis antibodies measured by a line blot assay in a broad spectrum of ILD.

Antibodies specific for t-RNA synthetases have been thoroughly described in myopathies and CTD-ILDs, but were also identified in other ILDs including IPF [3,4,5,6,11,12,13,14,15,16,17,18,19,20,21,22,23,24,25]. Ha antibodies are seen in myopathies and in 40% of Ssc [28,35,36]. We demonstrated novel data concerning the presence of anti-Ha in a broad spectrum of ILD. Interestingly, most subjects had a preserved pulmonary function and included patients with an unclassifiable IIP, characterized by a radiological and/or histopathological pattern alternative for UIP. Possibly, these patients may have a phenotype that is characterized by a mild disease course.

Anti-Ks has been described in 0.3–7% of ILD patients [33,34]. Interestingly, 70% had an IP without underlying CTD [33,34,41], which is agreement with 80% of anti-Ks positivity found our non-CTD-ILDs. Radiological and/or histological patterns of NSIP and OP (range 6–85.7%) have been described in ILD with anti-Ks [32,41,42,43] and other anti-t-RNA synthetases [11,16,23,25]. These results are in congruence with the presence of non-UIP patterns in our study. Strikingly, COP patients demonstrated no reactivity against Ks, nor against any other antigen. We found more UIP patterns on HRCT (35.7%) but less in lung biopsies (16.7%) compared to respectively 5% and 80% found in small ILD studies [42,43]. These results may be caused by the difference in study size and the absence of diagnostic lung biopsies in case of a typical radiological UIP.

A prevalence of 0.3% anti-Zo was found in ASS, of which 78% had an ILD [29,44]. Our assay identified antibodies against the alpha unit of Zo (Zoα) in 1.4% of the ILD cases, including ASS. We demonstrated novel associations of anti-Zoα with CTD-ILD and idiopathic IPs. Radiological patterns of UIP (14%) and NSIP with OP (range 14–57%) have been described [29], which is in line with prevalence of UIP and non-UIP patterns in our study. Interestingly, 66% of anti-Zo positive ASS showed reactivity against anti-Ro52 [29]. It is known that patients with both Ro52 and t-RNA synthase antibodies are characterized by chronic and severe ILD [6,19]. Possibly, patients with combined Ro52 and Zoα antibodies show similar clinical outcomes.

Antibodies against cN1A were described in IBM, PM/DM, Ssc, SLE, and Sjögren’s syndrome (range 0–37%). However, associations with ILD have not been identified [26,27,30,31,32,43,45]. We demonstrated novel data regarding the presence of cN1A antibodies in ILDs, including ASS, HP, and IPF. Moreover, various radiological and histological patterns can be seen, including UIP. A predominance for female patients was observed, which is in agreement with a study with DM patients [27]. In contrast, anti-cN1A positive patients with IBM, SLE, and Sjögren’s syndrome were mainly males [27,35,46].

This study was performed with patients who were all diagnosed by a standardized multidisciplinary approach in a tertiary ILD center in the Netherlands. It is the first study to describe prevalence and clinical features of novel myositis antibodies in a large ILD cohort compared to healthy controls. This retrospective study has an important limitation, as a potential selection bias of more severely impaired patients with pulmonary fibrosis is possible due to the patient population in a referral center. However, we do not expect this to have any major impact on the distribution of autoantibodies. Furthermore, the line-blot used in this study has been used for research purposes only to date. Thus, validation for implementation is not complete yet. In time, the results of our study will contribute to final implementation of these antibodies for clinical use. A selection bias of patients who underwent surgical lung biopsies is possible, as subjects with a (probable) UIP pattern on HRCT might not undergo surgery for diagnostic purposes. Furthermore, the prevalence of antibody reactivity was higher in ILD patients compared to healthy subjects, but statistical differences were not observed in terms of prevalence per antibody. This result is probably due to the relatively low prevalence found per antibody.

The findings of this study raise the question why antibodies are present in idiopathic IP, including IPF. An IP can occur two years before an associated CTD [3,6], but antibodies are present in true idiopathic IP as well [11,16,23,24,25]. In several studies, autoantibody producing plasma cells were identified in fibrotic lung tissue [47]. Furthermore, T follicular helper cells, which induce the production of antigen-specific antibodies in germinal centers, were increased and activated in the peripheral blood of patients with IPF compared to healthy controls [48]. Possibly, antibodies in idiopathic IP are randomly autoreactive and continuously generated at a certain stage of disease, without resulting in pathological autoimmunity as observed in CTD-ILDs. However, targets of these autoantibodies might actually participate in the disease process, culminating in pulmonary fibrosis. Although in general, the treatment response for immunosuppressive drugs is better in CTD-ILDs compared with other ILDs [5,6,7,8,9,10], one can speculate whether specific treatment regiments should be reconsidered in antibody positive ILD without established CTD. Recently, the use of anti-fibrotic therapy has been successfully demonstrated in Ssc-IP and progressive fibrosing ILDs [49,50]. It will be of interest to evaluate whether autoantibody positive idiopathic IP benefits from combining anti-fibrotic therapy with B cell targeted therapy when compared with antibody negative idiopathic IP. Such a study will benefit from additional serological parameters to signal immune activation status to determine whether ILD progression and autoantibody detection is paralleled by an ongoing immune response [51,52]. However, these studies may be difficult to realize because immunosuppressants can have a harmful effect in IPF in general [53,54].

In conclusion, our results contribute to the awareness that autoantibodies can be found in an IP without established CTD. Screening for antibodies on a regular basis could contribute to the identification of merely progressive fibrotic phenotype from those in which an ongoing autoimmune response which potentially feeds the fibrotic phenotype. A prospective cohort evaluation is needed to determine whether antibody positive idiopathic IP develop features of an associated CTD. Furthermore, it will be of interest to investigate associations between these novel antibodies with other myositis antibodies and treatment outcomes.

## Figures and Tables

**Table 1:** Baseline characteristics of patients with ILD.

Subjects.				
	All	CTD-ILD ^a^	Non-CTD-ILD ^b^	P ^g^
N	1194	131	1063	
Age (y)	65.1 (11.2)	60.1 (11.4)	65.7 (11.0)	<0.001
Sex (m), %	773 (64.7)	73 (55.7)	700 (65.9)	0.022
History of smoking, %	801 (67.1)	69 (52.7)	732 (68.9)	0.073
**Pulmonary function test ^c^**				
FVC (% pred)	80.6 (21.6)	80.4 (23.9)	80.6 (21.3)	0.940
FEV1 (% pred)	82.8 (21.2)	80.3 (22.9)	83.2 (21.0)	0.248
Dlco (% pred)	46.1 (15.8)	49.4 (17.2)	45.6 (15.7)	0.057
**HRCT scan ^d^**				
UIP	345 (29.9)	23 (18.7)	322 (31.3)	0.005
Probable UIP	172 (14.9)	13 (10.6)	159 (15.4)	0.152
Indeterminate	233 (20.2)	37 (30.1)	196 (19.0)	0.004
Alternative	403 (35.0)	50 (40.7)	353 (34.3)	0.161
**Histopathology ^e^**				
UIP	125 (34.8)	3 (9.1)	122 (37.4)	0.001
Probable UIP	15 (4.2)	2 (6.1)	13 (4.0)	0.573
Indeterminate	50 (13.9)	9 (27.3)	41 (12.6)	0.020
Alternative	169 (47.1)	19 (57.6)	150 (46.0)	0.205
ANA (%) ^**f**^	138 (18.2)	31 (36.4)	107 (10.1)	<0.001

Data are expressed as mean and standard deviation or numbers and percentage within the diagnosis group. FVC = forced vital capacity, expressed in percentage of predicted; FEV1 = forced expiratory volume in 1 s, expressed in percentage of predicted; Dlco = Diffusing capacity of the lung for carbon monoxide; UIP = usual interstitial pneumonia; ^a^ CTD-ILD = connective tissue disease related interstitial lung disease; ^b^ non-CTD-ILD = ILD without established CTD; ^c^ n = 918; ^d^ n = 1153; ^e^ n = 359; ^f^ ANA = antinuclear antibody, expressed as % positive; n = 757; ^g^ p < 0.05, differences between CTD-ILD and non-CTD-ILD patients are calculated by a two-side unpaired T-test for continuous variables or Chi-Square test for dichotomous variables.

**Table 2:** Prevalence of novel myositis autoantibodies in patients with ILD.

Antibody	N (%)					
	All ILD	CTD-ILD ^a^	Non-CTD-ILD ^b^	P ^c^	Healthy Controls	P ^d^
N	1194	131	1063		116	
Novel antibodies (p)	63 (5.3)	11 (8.4)	52 (4.9)	0.090	1 (0.9)	0.035
Novel antibodies (p+wp)	119 (10)	15 (11.5)	104 (9.8)	0.548	3 (2.6)	0.009
Ks (p)	15 (1.3)	3 (2.3)	12 (1.1)	0.222	-	0.388
Ks (p+wp)	24 (2.1)	3 (2.3)	21 (2.0)	0.741	-	0.262
Ha (p)	24 (2.0)	4 (3.1)	20 (1.9)	0.325	-	0.262
Ha (p+wp)	48 (4.0)	5 (3.8)	43 (4.0)	>0.999	1 (0.9)	0.119
Zoα (p)	17 (1.4)	4 (3.1)	13 (1.2)	0.106	-	0.390
Zoα (p+wp)	35 (2.9)	8 (6.1)	27 (2.5)	0.047	2 (1.7)	0.766
cN1A (p)	11 (0.9)	2 (1.5)	9 (0.8)	0.344	1 (0.9)	>0.999
cN1A (p+wp)	22 (1.8)	3 (2.3)	19 (1.5)	0.726	1 (0.9)	0.714

Data are expressed as numbers and percentage within the diagnosis group. (p) = positive level (wp) = weak positive level; ^a^ CTD-ILD = connective tissue disease related interstitial lung disease; ^b^ non-CTD-ILD = ILD without established CTD. ^c^ *p* < 0.05, considered significant; differences in frequencies between CTD-ILD and non-CTD-ILD patients, calculated by a Chi-Square or Fisher’s exact test for dichotomous variables. ^d^ *p* < 0.05, considered significant; differences in frequencies between all ILD patients and healthy controls, calculated by a Chi-Square or Fisher’s exact test for dichotomous variables.

**Table 3:** Characteristics of ILD patients with and without novel autoantibody reactivity.

	Novel Autoantibody Positive	Novel Autoantibody Negative	*p*-Value ^d^
N	63	1075	
Age (y)	64.6 (11.5)	65.1 (11.1)	0.713
Sex (m), %	33 (52.4)	699 (65.0)	0.042
History of smoking, %	39 (61.9)	722 (67.2)	0.557
**Pulmonary function test**			
FVC (% pred)	82.1 (20.8)	81.9 (34.6)	0.967
FEV1 (% pred)	84.6 (20.7)	84.4 (35.5)	0.981
Dlco (% pred)	47.4 (17.5)	46.0 (15.8)	0.607
**HRCT scan ^a^**			
UIP	13 (22.0)	321 (30.9)	0.158
Probable UIP	9 (15.3)	154 (14.8)	0.928
Indeterminate UIP	15 (25.4)	202 (19.4)	0.262
Alternative	22 (37.3)	362 (34.8)	0.701
**Histopathology ^b^**			
UIP	2 (11.1)	117 (35.8)	0.032
Probable UIP	1 (5.6)	14 (4.3)	0.560
Indeterminate UIP	3 (16.7)	44 (13.5)	0.722
Alternative	12 (66.7)	152 (46.5)	0.095
ANA (%) ^c^	10 (24.4)	129 (17.9)	0.293

Data are expressed as mean and standard deviation or numbers and percentage per group. Included antibodies: anti-Ks, anti-Ha, anti-Zoα, anti-cN1A; on a positive level (antibody positive level; antibody reactivity on weakly positive level excluded) or negative level (antibody negative); FVC = forced vital capacity, expressed in percentage of predicted; FEV1 = forced expiratory volume in 1 s, expressed in percentage of predicted; Dlco = Diffusing capacity of the lung for carbon monoxide; UIP = usual interstitial pneumonia. ^a^ myositis antibody positive ILD: n = 59; myositis antibody negative ILD: n = 1039; ^b^ myositis antibody positive ILD: n = 18; myositis antibody negative ILD: n =327; ^c^ ANA = antinuclear antibody, expressed as % positive; myositis antibody positive ILD n = 41; myositis antibody negative ILD n = 716; ^d^ *p* < 0.05, differences between the groups calculated by a two-sided sample T-test for continuous variables or Chi-Square or Fisher’s exact test for dichotomous variables.

**Table 4:** Characteristics of ILD patients with positive novel antibody reactivity.

	Autoantibody				
	Ha	Ks	Zoα	cN1A	*p*-Value ^d^
N	24	15	17	11	
Age (y)	68.3 (11.3)	63.5 (12.0)	62.9 (12.9)	59.8 (7.3)	0.529
Sex (m), %	14 (58.3)	8 (53.3)	11 (64.7)	2 (18.2)	0.082
History of smoking, %	16 (66.7)	7 (46.7)	12 (75.0)	6 (54.5)	0.584
**Pulmonary function test**					
FVC (% pred)	83.3 (25.0)	81.0 (16.0)	77.6 (19.6)	76.5 (15.0)	0.984
FEV1 (% pred)	90.8 (26.5)	83.9 (15.6)	77.5 (15.8)	75.0 (14.6)	0.548
Dlco (% pred)	48.9 (16.0)	49.6 (17.6)	41.9 (13.4)	40.3 (16.8)	0.689
**HRCT scan ^a^**					
UIP	5 (21.7%)	5 (35.7%)	2 (12.5%)	4 (40.0%)	0.048
Probable UIP	4 (17.4%)	3 (21.4%)	2 (12.5%)	-	0.278
Indeterminate UIP	6 (26.1%)	3 (14.3%)	5 (31.3%)	2 (20.0%)	0.652
Alternative	8 (34.8%)	4 (28.6%)	7 (43.8%)	4 (40.0%)	0.694
**Histopathology** ^b^					
UIP	-	1 (16.7%)	-	1 (20.0%)	0.542
Probable UIP	-	-	-	1 (20.0%)	0.437
Indeterminate UIP	1 (25.0)	1 (16.7%)	-	1 (20.0%)	0.727
Alternative	3 (75.0)	4 (66.7%)	3 (100%)	2 (40.0%)	0.256
ANA (%) ^c^	4 (23.5)	1 (10.0)	2 (16.7)	3 (50.0)	0.303

Data are expressed as mean and standard deviation or numbers and percentage per positive myositis antibody group; FVC = forced vital capacity, expressed in percentage of predicted; FEV1 = forced expiratory volume in 1 s, expressed in percentage of predicted; Dlco = Diffusing capacity of the lung for carbon monoxide; UIP = usual interstitial pneumonia. ^a^ Number of patients with available high resolution computed tomography (HRCT) scan: Ha (n = 23); Ks (n = 15); Zoα (n = 16); cN1A (n = 10); ^b^ Number of patients with available histopathological lung biopsies: Ha (n = 4); Ks (n = 6); Zoα (n = 3); cN1A (n = 5); ^c^ Number of patients with available antinuclear antibody (ANA): Ha (n = 17); Ks (n = 10); Zoα (n = 12); cN1A (n = 6) ^d^ p < 0.05, differences between the myositis antibodies calculated by a one way ANOVA for continuous variables or Chi-Square or Fisher’s exact test for dichotomous variables.

**Table 5:** Associations of novel myositis antibodies in ILD with and without established CTD.

Antibody	CTD-ILD (n = 131)			Non-CTD-ILD (n = 1063)					
	Number Neg	Number Weak pos	Number Pos	Number Neg	Number Weak pos	Number Pos	OR p OR wp OR wp+p ^a^	95% CI ^b^	P ^c^
Ks	128	-	3	1042	9	12	2.04	0.57–7.31	0.276
							∞	∞	∞
							0.81	0.34–3.95	0.809
Ha	126	1	4	1020	23	20	1.62	0.55–4.81	0.386
							0.35	0.05-2.63	0.309
							0.94	0.37–2.42	0.900
Zoα	123	4	4	1036	14	13	2.59	0.83–8.07	0.100
							2.41	0,78–7.43	0.127
							2.50	1.11–5.61	0.027
cN1A	128	1	2	1044	10	9	1.81	0.39–8.48	0.450
							0.82	0.10–6.42	0.847
							1.29	0.38–4.41	0.687

CTD-ILD = connective tissue disease related interstitial lung disease; non-CTD-ILD = ILD without established CTD. ^a^ OR: odds ratio for positive level (OR p); odds ratio for weak positive level (OR wp); odds ratio for weak positive level + positive level (OR wp+p); ^b^ 95% confidence interval of odds ratio’s; ^c^ Logistic regression analysis of CTD-ILD versus no established CTD-ILD (non-CTD-ILD) patients with positive, weak positive and negative antibody, with predicted probability for CTD-ILD.

## Supplementary Materials

The following are available online at https://www.mdpi.com/2077-0383/9/9/2944/s1, Table S1: Baseline characteristics of CTD-ILD patients, Table S2: Baseline characteristics of patients with ILD without established CTD, Table S3: Prevalence of novel myositis antibodies in CTD-ILD patients. Table S4: Prevalence of novel myositis antibodies in patients with an ILD without established CTD
