# Supplementary material for: Prevalence of Novel Myositis Autoantibodies in a Large Cohort of Patients with Interstitial Lung Disease
Source: J Clin Med. 2020 Sep 11;9(9):2944. doi: 10.3390/jcm9092944 (PMC7563342; doi:10.3390/jcm9092944)
Supplement: Supplementary file 1 [file jcm-09-02944-s001.pdf]

Supplementary data

Supplementary Table S1. Baseline characteristics of CTD-ILD patients.

Subjects	All CTD-ILD	ASS	Sjögren's syndrome	RA-ILD	Ssc	PM/DM	IBM	SLE	Mixed CTD-ILD	Other CTD-ILD [a]	P[f]
N	131	23	24	44	16	3	1	1	15	4	
Age (y)	60.1 (11.4)	57.4 (9.9)	58.8 (15.2)	64.6 (7.9)	58.0 (9.3)	54.0 (5.4)	69.7 (-)	68.3 (-)	55.0 (14.1)	61.7 (15.5)	0.072
Sex (m), %	73 (55.7)	12 (52.2)	4 (16.7)	32 (72.7)	9 (56.3)	2 (66.7)	1 (100.0)	1 (100.0)	8 (53.3)	4 (100.0)	<0.001
History of smoking, %	69 (52.7)	10 (43.5)	9 (37.5)	27 (61,4)	10 (62.5)	2 (66.7)	-	1 (100.0)	8 (53.3)	2 (50.0)	0.129
**Pulmonary function test [b]**											
FVC (% pred)	80.4 (23.9)	72.3 (23.5)	82.3 (24.8)	80.1 (23.5)	89.9 (27.3)	77.2 (29.4)	96.7 (-)	99.0 (-)	82.6 (23.1)	77.9 (17.8)	0.697
FEV1 (% pred)	80.3 (22.9)	70.7 (20.1)	81.9 (23.1)	83.3 (25.1)	88.5 (25.8)	73.8 (21.8)	97.6 (-)	81.0 (-)	78.8 (19.3)	76.8 (21.7)	0.636
Dlco (% pred)	49.4 (17.2)	42.6 (14.1)	52.0 (15.1)	48.8 (17.2)	48.9 (17.4)	46.0 (36.6)	67.5 (-)	56.0 (-)	60.5 (17.7)	45.5 (20.9)	0.437
**HRCT scan[c]**											
UIP	23 (18.7)	1 (5.0)	1 (4.2)	17 (41.5)	1 (6.7)	1 (33.3)	-	-	2 (14.3)	-	0.002
Probable UIP	13 (10.6)	-	4 (16.7)	6 (14.6)	2 (13.3)	-	-	-	1 (7.1)	-	0.409
Indeterminate	37 (30.1)	5 (25.0)	7 (29.2)	10 (24.4)	6 (40.0)	1 (33.3)	1 (100.0)	1 (100.0)	5 (35.7)	1 (25.0)	0.573
Alternative	50 (40.7)	14 (70.0)	12 (50.0)	8 (19.5)	6 (40.0)	1 (33.3)	-	-	6 (42.9)	3 (75.0)	0.009
**Histopathology [d]**											
UIP	3 (9.1)	-	-	2 (33.3)	1 (33.3)	-	-	-	-	-	0.124
Probable UIP	2 (6.1)	1 (20.0)	1 (8.3)	-	-	-	-	-	-	-	0.669
Indeterminate	9 (27.3)	1 (20.0)	4 (33.3)	2 (33.3)	-	-	-	-	1 (20.0)	1 (50.0)	0.703
Alternative	19 (57.6)	3 (60.0)	7 (58.3)	2 (33.3)	2 (66.7)	-	-	-	4 (80.0)	1 (50.0)	0.743
ANA (%) [e]	31 (36.4)	6 (42.9)	12 (60.0)	2 (7.4)	3 (50.0)	-	-	-	7 (63.6)	1 (25.0)	0.001

Data are expressed as mean and standard deviation or numbers and percentage within the diagnosis group. CTD-ILD = connective tissue disease related interstitial lung disease; HRCT = high resolution computed tomography; UIP = usual interstitial pneumonia; FVC = forced vital capacity, expressed in percentage of predicted; FEV1 = forced expiratory volume in 1 second, expressed in percentage of predicted; Dlco = Diffusing capacity of the lung for carbon monoxide; ANA = antinuclear antibody, expressed as % positive; UIP = usual interstitial pneumonia; ASS = antisynthetase syndrome; RA-ILD = rheumatoid arthritis associated interstitial lung disease; Ssc = systemic sclerosis; PM/DM = polymyositis/dermatomyositis; IBM = inclusion body myositis; SLE = systemic lupus erythematosus. a Other CTD-ILD: IgG4 related disease (n = 3), Bechterew's disease (n = 1) b n = 107 c n = 123 d n = 33 e n = 82; f $p < 0.05$, differences between the CTD-ILD subgroups are calculated by a one way ANOVA for continuous variables or Chi-Square or Fisher's exact test for dichotomous variables.

Supplementary Table S2. Baseline characteristics of patients with ILD without established CTD.

**Subjects**	**Non-CTD-ILD**	**IPF**	**Unclassifiable IIP**	**HP**	**NSIP**	**COP**	**Pneumoconiosis**	**Drug induced**	**Other ILD** [b]	**p** [g]
N	1063	301	390	200	66	31	13	12	50	
Age (y)	65.7 (11.0)	67.3 (10.0)	67.4 (10.3)	62.3 (11.5)	64.9 (11.0)	65.0 (7.0)	71.1 (10.1)	63.1 (18.1)	57.3 (12.0)	<0.001
Sex (m), %	700 (65.9)	240 (79.7)	261 (66.9)	94 (47.0)	38 (57.6)	19 (61.3)	11 (84.6)	4 (41.7)	32 (64.0)	<0.001
History of smoking, %	732 (68.9)	239 (79.4)	268 (68.7)	112 (56.0)	38 (57.6)	21 (67.7)	10 (76.9)	7 (58.3)	37 (74.0)	<0.001
**Pulmonary function test** [c]										
FVC (% pred)	80.6 (21.3)	79.3 (20.1)	80.3 (21.3)	77.9 (20.1)	85.4 (25.0)	100.4 (21.0)	81.1 (22.7)	75.5 (16.5)	86.4 (22.2)	<0.001
FEV1 (% pred)	83.2 (21.0)	83.7 (19.3)	84.2 (20.6)	78.3 (20.4)	88.1 (27.1)	96.3 (22.0)	82.3 (22.2)	75.6 (15.7)	78.3 (23.7)	0.003
Dlco (% pred)	45.6 (15.7)	42.4 (12.5)	46.1 (16.3)	45.3 (14.6)	46.5 (15.2)	71.6 (16.6)	54.9 (18.7)	54.0 (14.1)	44.2 (18.8)	<0.001
**HRCT scan** [d]										
UIP	322 (31.3)	248 (83.2)	44 (11.4)	26 (13.5)	1 (1.6)	-	2 (16.7)	-	1 (2.1)	<0.001
Probable UIP	159 (15.4)	31 (10.4)	110 (28.5)	8 (4.2)	10 (16.4)	-	-	-	-	<0.001
Indeterminate	196 (19.0)	17 (5.7)	102 (26.4)	30 (15.6)	23 (37.7)	2 (8.3)	2 (16.7)	2 (20.0)	18 (38.3)	<0.001
Alternative	353 (34.3)	2 (0.7)	130 (33.7)	128 (66.7)	27 (44.3)	22 (91.7)	8 (66.7)	8 (100.0)	28 (59.6)	<0.001
**Histopathology** [e]										
UIP	122 (37.4)	70 (94.6)	45 (43.7)	6 (7.8)	-	-	-	-	1 (4.0)	<0.001
Probable UIP	13 (4.0)	2 (2.7)	9 (8.7)	2 (2.6)	-	-	-	-	-	0.128
Indeterminate	41 (12.6)	2 (2.7)	18 (17.5)	8 (10.4)	6 (24.0)	3 (15.8)	-	-	4 (16.0)	0.032
Alternative	150 (46.0)	-	31 (30.1)	61 (79.2)	19 (76.0)	16 (84.2)	1 (100.0)	2 (100.0)	20 (80.0)	<0.001
ANA (%) [f]	107 (15.9)	17 (10.2)	59 (21.1)	17 (14.0)	6 (13.6)	2 (13.3)	-	-	6 (18.8)	0.023

Data are expressed as mean and standard deviation or numbers and percentage within the diagnosis group. non-CTD-ILD = ILD without established CTD; IPF = idiopathic pulmonary fibrosis; unclassifiable IIP = unclassifiable idiopathic interstitial pneumonia; HP = hypersensitivity pneumonitis; NSIP = non-specific interstitial pneumonia; COP = cryptogenic organizing pneumonia; HRCT = high resolution computed tomography; ANA = antinuclear antibody, % positive; UIP = usual interstitial pneumonia; FVC = forced vital capacity, expressed in percentage of predicted; FEV1 = forced expiratory volume in 1 second, expressed in percentage of predicted; Dlco = Diffusing capacity of the lung for carbon monoxide; UIP = usual interstitial pneumonia. Other ILD: desquamative interstitial pneumonia (n = 21) respiratory bronchiolitis interstitial pneumonia (n = 10) , smoking-related interstitial pneumonia (n = 6), combined pulmonary fibrosis and emphysema (n = 4), vasculitis (n = 2), bronchiolocentric interstitial pneumonia (n = 2), pleuroparenchymal fibroelastosis (n = 2), chronic eosinophilic pneumonia (n = 1), diffuse alveolar haemorrhage with amyloidosis (n = 1), diffuse alveolar damage (n = 1). c n = 912; d n = 1030; e n = 326; f n = 675 g $p < 0.05$, differences between the non-CTD-ILD subgroups are calculated by a one way ANOVA for continuous variables or Chi-Square or Fisher's exact test for dichotomous variables.

Supplementary Table S3: Prevalence of novel myositis antibodies in CTD-ILD patients.

	N (%)									
	**All CTD-ILD**	**ASS**	**Sjögren's syndrome**	**RA-ILD**	**Ssc**	**PM/DM**	**IBM**	**SLE**	**Mixed CTD-ILD**	**Other CTD-ILD** [a]
N	131	23	24	44	16	3	1	1	15	4
Ks (p)	3 (2.3)	-	1 (4.2)	1 (2.3)	-	-	1 (100)	-	-	-
Ks (p+wp)	3 (2.3)	-	1 (4.2)	1 (2.3)	-	-	1 (100)	-	-	-
Ha (p)	4 (3.1)	1 (4.3)	2 (8.3)	-	1 (6.3)	-	-	-		-
Ha (p+wp)	5 (3.8)	1 (4.3)	2 (8.3)	-	1 (6.3)	-	-	-	1 (6.7)	-
Zoα (p)	4 (3.1)	2 (8.7)	1 (4.2)		-	1 (33.3)	-	-	-	-
Zoα (p+wp)	8 (6.1)	2 (8.7)	2 (8.3)	2 (4.5)	-	1 (33.3)	-	-	-	-
cN1A (p)	2 (1.5)	2 (8.7)	-	-	-	-	-	-	-	
cN1A (p+wp)	3 (2.3)	2 (8.7)	-	-	-	-	-	-	-	1 (25.0)

Data are expressed as numbers and percentage within the diagnosis group. (p) = positive level (wp) = weak positive level CTD-ILD = connective tissue disease related interstitial lung disease; ASS: anti-synthetase syndrome; RA-ILD; rheumatoid arthritis associated interstitial lung disease; Ssc; systemic sclerosis (n = 16), PM/DM; polymyositis/dermatomyositis; IBM; inclusion body myositis; SLE; systemic lupus erythematosus. a Other CTD-ILD: IgG4 related disease (n = 3), Bechterew's disease (n = 1).

Supplementary Table S4: Prevalence of novel myositis antibodies in patients with an ILD without established CTD.

	N (%)								
	**Non-CTD-ILD**	**IPF**	**Unclassifiable IIP**	**HP**	**NSIP**	**COP**	**Pneumoconiosis**	**Drug induced**	**Other ILD [b]**
N	1063	301	390	200	66	31	13	12	50
Ks (p)	12 (1.1)	1 (0.3)	5 (1.3)	3 (1.5)	2 (3.0)	-	-	-	1 (2.0)
Ks (p+wp)	21 (2.0)	3 (0.7)	10 (1.3)	3	4 (3.0)	-	-	-	1
Ha (p)	20 (1.9)	3 (1.0)	10 (2.6)	6 (3.0)	-	-	1 (7.7)	-	-
Ha (p+wp)	43 (4.0)	10 (2.3)	18 (2.1)	13 (3.5)	1 (1.5)	-	1	-	-
Zoα (p)	13 (1.2)	1 (0.3)	6 (1.5)	2 (1.0)	1 (1.5)	-	-	1 (8.3)	2 (4.0)
Zoα (p+wp)	27 (2.5)	5 (1.3)	12 (1.5)	5 (1.5)	1	-	-	1	3 (2.0)
cN1A (p)	9 (0.8)	2 (0.7)	1 (0.3)	3 (1.5)	1 (1.5)	-	-	-	2 (4.0)
cN1A (p+wp)	19 (1.5)	3 (0.3)	5 (1.0)	8 (2.5)	1	-	-	-	2

Data are expressed as numbers and percentage within the diagnosis group. (p) = positive level (wp) = weak positive level. no established CTD-ILD (non-CTD-ILD); IPF = idiopathic pulmonary fibrosis; Unclassifiable IIP = unclassifiable idiopathic interstitial pneumonia; HP = hypersensitivity pneumonitis; NSIP = non-specific interstitial pneumonia; COP = cryptogenic organizing pneumonia. Other ILD: desquamative interstitial pneumonia (n = 21) respiratory bronchiolitis interstitial pneumonia (n = 10) , smoking-related interstitial pneumonia (n = 6), combined pulmonary fibrosis and emphysema (n = 4), vasculitis (n = 2), bronchiolocentric interstitial pneumonia (n = 2), pleuroparenchymal fibroelastosis (n = 2), chronic eosinophilic pneumonia (n = 1), diffuse alveolar haemorrhage with amyloidosis (n = 1), diffuse alveolar damage (n = 1).
